# Transfection of STAT3 overexpression plasmid mediated through recombinant lentivirus promotes differentiation of bone marrow mesenchymal stem cells into neural cells in fetal rats with spina bifida aperta

**DOI:** 10.18632/aging.203524

**Published:** 2021-09-14

**Authors:** Mingyu Jiang, Jiale Feng, Rong Fu, Yanbo Pan, Xu Liu, Jicheng Dai, Chunming Jiang, Yunpeng Hao, Mingyong Ren

**Affiliations:** 1 Department of Pediatrics, The First Affiliated Hospital of Harbin Medical University, Harbin 150001, P.R. China; 2 Department of Ultrasound Medicine, The Fifth Hospital of Harbin, Harbin 150040, P.R. China; 3 Department of Neurosurgery, Tieling Central Hospital, Tieling 112000, P.R. China; 4 Department of Stomatology, The Fourth Affiliated Hospital of Harbin Medical University, Harbin 150001, P.R. China; 5 Department of Pediatrics, The First Hospital of Jilin University, Changchun 130021, P.R. China

**Keywords:** bone marrow mesenchymal stem cells, spina bifida aperta, signal transducer and activator of transcription-3, lentivirus transfection, nerve cells

## Abstract

We investigated the influence of signal transducer and activator of transcription-3 (STAT3) on the spinal cord tissue grafts of rat fetuses with spina bifida aperta. In particular, we hoped to identify whether transfection of the STAT3 overexpression plasmid increases the survival of spinal cord transplantation in order to improve therapeutic efficacy. The fetal rat model of spina bifida aperta was established using retinoic acid and treated with a microsurgical injection of bone marrow mesenchymal stem cells (BMSCs). The animals were divided into either the blank control group, negative control group or the experimental group. The optical density (OD) value of BMSCs viability was determined using the Cell Counting Kit-8 (CCK-8). The expression of STAT3, phosphorylated STAT3 (pSTAT3), neural markers and apoptosis-related factors were evaluated using real-time PCR and Western blot. The OD value in the experimental group was highest at eight hours after transplantation using CCK-8. The expression of pSTAT3, glial fibrillary acidic protein, neuron-specific enolase, neurofilament and nestin in the experimental group was significantly higher compared to the blank control group and negative control group (*P*<0.05). However, STAT3 expression in the experimental group was statistically significantly decreased (*P<*0.05). The relative expression of caspase-8 and bcl-2 in the experimental group were significantly lower compared to the blank control group and negative control group (*P*<0.05). Transfection of the recombinant lentivirus-mediated STAT3 overexpression plasmid with BMSCs can help improve the efficiency of transforming into neural cells and provide new seed cells for the treatment of congenital spina bifida aperta.

## INTRODUCTION

Neural tube defects (NTDs) in children can result in severe birth abnormalities. The primary mechanism of NTDs is currently considered to be aberrant neural tube closure. Congenital spina bifida aperta (SBA) is the most prevalent of many kinds of NTDs [1]. SBA's origin and pathophysiology aren't completely understood. However, it is widely assumed that embryonic development is influenced by a range of aberrant gene regulation and adverse environmental variables [2]. Apoptosis is a critical process in the growth, maturation, and creation of the nervous system [3]. Many studies have shown that neuronal apoptosis is linked to neural tube deformation in children. The specific process, however, is unknown. Apoptosis happens at a precise time and location during normal embryonic development, according to developmental biology studies. If this carefully controlled, planned cell death is disrupted, it can result in a variety of congenital abnormalities [4].

SBA in children has no viable therapy at the moment. During the embryonic phase, fetuses with severe abnormalities die. Even if individuals with minor malformations survive, the majority of them have neurological problems, such as limb paralysis, defecation difficulties, and paraplegia [5]. It has the potential to negatively impact children's development and overall growth, as well as the quality of their life on the whole. However, in recent years, stem cell transplantation technology has given new hope to medical science for treating congenital nervous system (NS) malformations. Bone marrow mesenchymal stem cells (BMSCs) possess the capability of self-renewal and differentiation in several directions. BMSCs can develop into neural cells, myoblasts, adipocytes, chondrocytes, osteoblasts, and additional types of cells in response to physical, chemical, and cytokine stimulation. Under specific conditions or the microenvironment, they may also develop into endoderm cells, ectoderm cells, or mesoderm stromal cells, which is useful for treating nervous system degenerative disorders, congenital malformations, and stress damage [6, 7]. In gene therapy and bioremediation, this method is frequently used [8]. Because BMSCs are made up of basic materials, can be readily isolated and purified, and are generated from a patient's own body, the immunological exclusion is uncommon. As a result, in tissue engineering BMSCs can furnish a valuable supply of seed cells. The use of BMSCs as a therapy for SBA in children is expected to be a new technique [9].

STAT3, belonging to the STAT family, was discovered and isolated as an acute stage response factor (aprf) in 1994 [10]. Many growth factors and cytokines, including LIF, IL-6, EGF, CNTF, and Onco-statin M can activate STAT3. Recognizing the receptor complex, which was created utilizing the cell surface-bound glycoprotein gp130 on the cell surfaces and additional species including CNTFR and LIFR, triggered the STAT3 signal transduction pathway. Some scientists think that the synchronization of the STAT3 and MAPK signaling pathways is essential for stem cell self-renewing and differentiation [11]. As members of key signal transduction pathways in cells, both the STAT and MAPK families can bring about the transfer of stimulus from the extracellular surface to the nucleus. They also have a role in cell growth, development, differentiation, and death, among other physiological processes. Furthermore, the protein STAT3, not only engages in a variety of physiological events involved in nervous system development, but it also governs nerve cell survival and repair via the production of neuroregulatory cytokines [12]. A prominent member of the caspase group and a member of a group of aspartic acid-specific cysteine proteases, caspase-8, is involved in apoptosis execution and controls cell morphology and physicochemical changes [13]. Meanwhile, bcl-2 plays a role in apoptosis early-onset, signal transmission, and late effects [14]. Abberation of bcl-2 and caspase-8 expression can subsequently result in apoptosis and proliferation problems, which can lead to nervous system illnesses [15].

The entire bone marrow adherence technique was used to identify and purify BMSCs in this study. A recombinant lentivirus was used to transfect the STAT3 overexpression plasmid into BMSCs, and retinoic acid was used to generate a fetal rat model of SBA. By combining fetal surgery with microsurgery injection, the fetal rats were subjected to BMSC transplants. In an attempt to investigate the function of pSTAT3, STAT3, apoptosis-related factors (bcl-2 and caspase 8), and nerve markers (NF, NSE, GFAP, and nestin) in the transplantation therapy of SBA, researchers looked at the expression levels of pSTAT3, STAT3, nerve markers (nestin, NF, NSE, and GFAP) in the fetal rats’ spinal cords.

## RESULTS

### BMSCs were successfully identified using the flow cytometry technique in Wistar rats prior to transplantation

**Figure 1:**
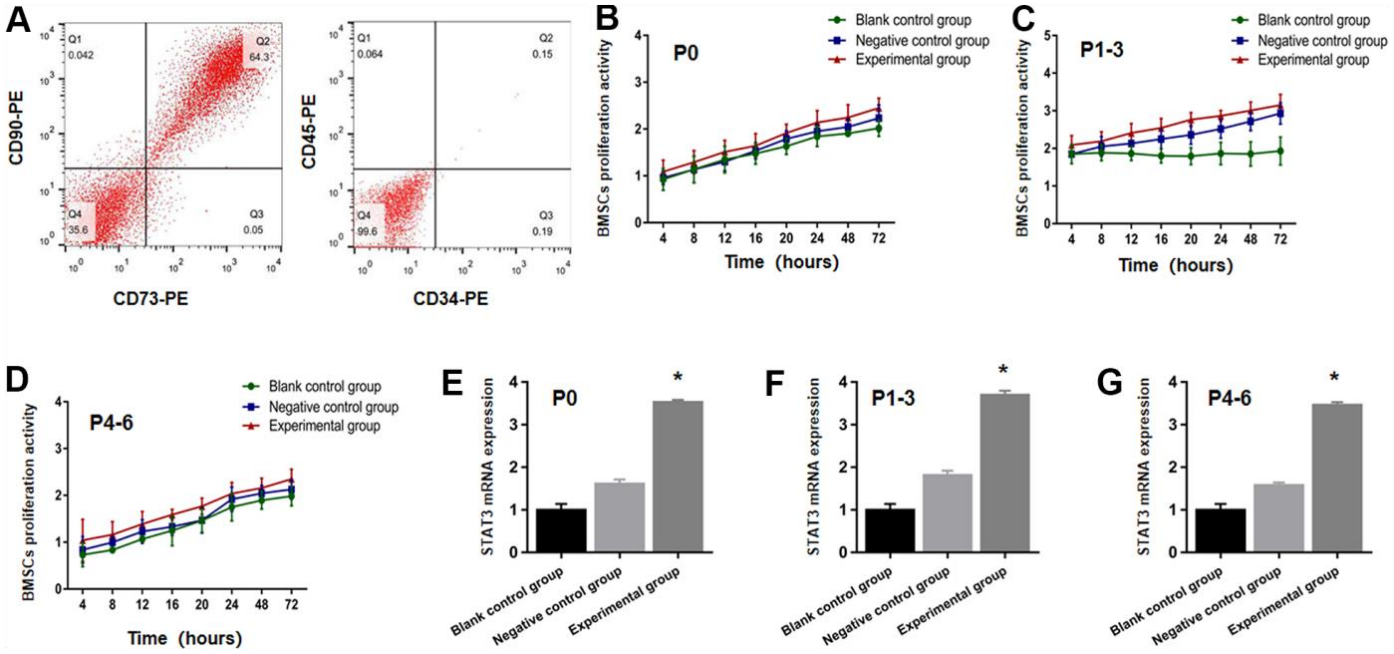
**STAT3 plasmid transfected BMSCs were successfully identified.** (**A**) The expression of CD34, CD45, CD73 and CD90 from BMSCs were detected through the use of flow cytometry. BMSCs proliferation activity at P0 (**B**), P1-3 (**C**), P4-6 (**D**) were determined using the CCK-8 test in the blank control group, negative control group and experimental group prior to transplantation. The STAT3 mRNA expression at P0 (**E**), P1-3 (**F**), P4-6 (**G**). The BMSCs in the experimental group increased more significantly than that in blank control group and negative control group. * *P* <0.05 versus blank control group + negative control group.

Flow cytometry was used to identify four different sorts of the markers in BMSC surface at P0-6. CD90 and CD73 were found to be positive, whereas CD45 and CD34 were found to be negative (Figure 1A). CD90 and CD73 were ribosome and fibrin receptors upon the BMSCs surface, respectively, and in terms of the cell, the origin was essentially non-hematopoietic. CD45 and CD34 were endothelial and hematopoietic cell surface receptors, indicating that Wistar BMSCs had been effectively isolated.

### CCK-8 assay for the assessment of effective transfection in the proliferative activity of BMSCs, and the STAT3 plasmid prior to transplantation

The BMSCs proliferative function at P0, P1-3, P4-6 in the experimental group, the group of negative control, and blank control group was investigated at 72, 48, 24, 20, 16, 12, 8, and 4 hours as time nodes prior to transplantation. As evident from Figure 1B–1D, the results demonstrated no significant difference with regards to the total count of viable cells in all the three groups at the time point (*P>*0.05), thereby implying that the proliferation of BMSCs is not affected by lentivirus transfection. It can be seen in Figures 1E–1G that the mRNA expressions of STAT3 in the experimental group rose significantly in comparison to the negative control and blank control groups (*P<*0.05), indicating that the transfection of plasmid with STAT3 overexpression was effective.

### Survival of rat fetuses in individual groups following the transplantation of BMSCs

BMSCs were implanted into 252 fetuses from 72 pregnant rats. On the 18th day following transplantation, five pregnant rats died, making the rate of survival for transplanted pregnant rats 93% (67/72). A total of 252 transplanted fetuses were extracted, with 210 (83.3%) of them surviving. On E18, E19, E19.5, and E20, the pregnant, transplanted rats were killed, and the transplanted fetuses with SBA were gathered for a samples collection from the lumbosacral region of the spine. In total, 70 cases arose from BMSCs that had not been transfected with recombinant lentivirus (blank control group), 74 cases from BMSCs that had been transfected with the plasmid with STAT3 overexpression (experimental group), and 76 cases from BMSCs that had only been transfected with recombinant lentivirus (negative control group).

### BMSCs in combination with STAT3 overexpression plasmid transfection are able to enhance the transplantation achievement rate

**Figure 2:**
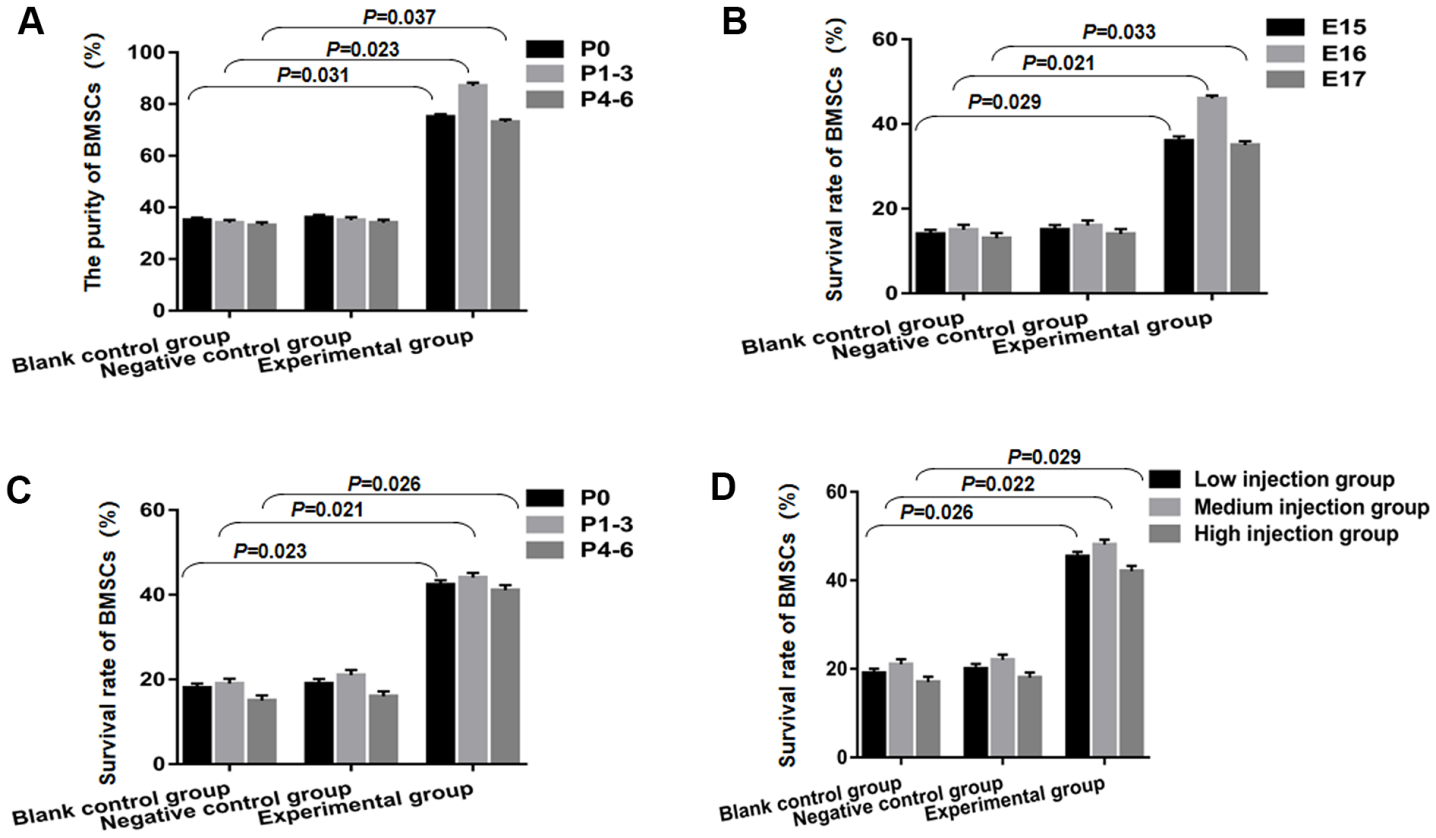
**BMSCs combined with STAT3 overexpression plasmid transfection can improve the success rate of transplantation.** (**A**) The purity of BMSCs in the experimental group was statistically significant, compared to the blank control group and negative control group. (**B**) The overall survival rate of BMSCs in the experimental group was found to be slightly higher than that of blank control group and negative control group from E15 to E17, and statistically significant. (**C**) BMSCs survival was notably increased at P0, P1-3 and P4-6 compared to the blank control group and negative control group (*P*<0.05). (**D**) The medium injection group was remarkably different from the change that occurred in the low injection group and high injection group on E19 fetuses after P1-3 BMSCs transplantation, which reached a statistical significance.

The purity of BMSCs, prior to transplantation was found to be enriched from 33±1.22% from P4 to P6, 34±1.16% at P1-3, and 35±1.02% at P0 in the blank control group. It was observed to be 36±0.98% at P0, 35±0.87% at P1-3, and 34±0.71% from P4 to P6 in the group of negative control. However, the purity of BMSCs enriched from 73±1.07% at P4 to P6, 87±1.29% from P1 to P3, and 75±1.13% from P0 among BMSCs combining with STAT3 overexpression plasmid transfection. CD90 and CD73 expression was also tested at these time points and were found to be statistically significant (*P<*0.05, in comparison to negative control and blank control groups, accordingly) as shown in Figure 2A. The purity of the P1-3 BMSCs coupled with STAT3 overexpression plasmid transfection was found to be higher than that of the P0 and P4-6 BMSCs. Then, on E15, E16, and E17, P1-3 BMSCs were transplanted into individual groups of rat fetuses to see if the transfection of plasmid with STAT3 overexpression affects the ratio of the survival of cells.

Green fluorescent protein (GFP) was used to label BMSCs, and on E19 positive cell counts were estimated following transplantation. In the experimental group, the overall survival rates of BMSCs on E15 were found to be 36±1.08%, 46 ±0.72% on E16, and 35±0.93% on E17. These were considerably high in comparison to the blank control and negative control groups, and also significant statistically as shown in Figure 2B (*P<*0.05). In the experimental groups accomplished on developing stages (E16) in comparison to the E17 and E15 groups, improved survival following STAT3 overexpression plasmid transfection was obtained, nevertheless, it has not approached statistical significance (*P*=0.32 E17 against E16; *P* =0.65 E15 against E16). Second, we sought to see if following STAT3 overexpression plasmid transfection, the survival ratio of various BMSC passages transplanted manifested any difference. As a result, we classified the three groups that received E16 from fetuses of rats into P0, P1-3, and P4-6. Within the experimental group belonging to the E19 transplanted fetuses, BMSCs survival was P0 (42.36±1.08%), P1-3 (44.06±1.1%), and P4-6 (41.09±1.2%), demonstrating a statistically significant increment in comparison to the negative control and blank control groups as depicted in Figure 2C (*P<*0.05). Finally, we looked at how the quantity of injected cells affected the ratio of survival when P1-3 BMSCs were transfected with a STAT3 overexpression plasmid and transplanted into E16 fetuses of rats. On the E19 transplanted fetuses, the medium injection group (2000–4000 cells/injection/spinal cord) differed substantially from the high injection group (4001–6000 cells/injection/spinal cord) and the low injection group (1000–1999 cells/injection/spinal cord) (*P<*0.05, Figure 2D).

### The CCK-8 test for detecting cell proliferation activity following BMSCs transplantation

**Figure 3:**
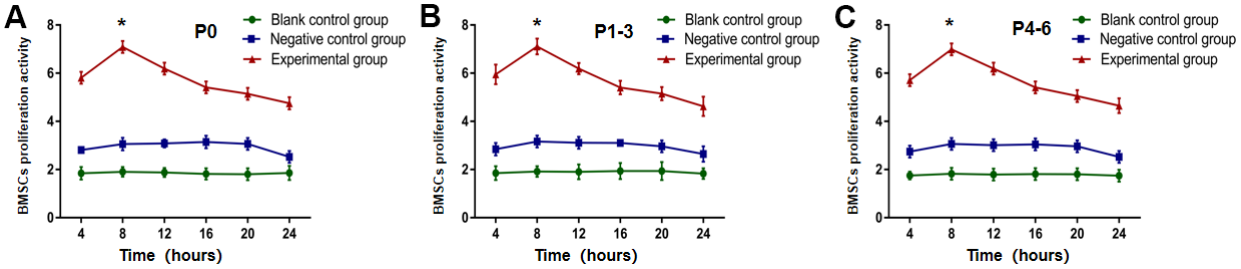
**OD value from P0-6 BMSCs in experimental group was highest at eight hours after E16 transplantation using the CCK-8 test.** The OD value from (**A**) P0, (**B**) P1-3 and (**C**) P4-6 in experimental group was the highest at eight hours after E16 transplantation, and then gradually decreased (*P*<0.05). * *P* <0.05 versus blank control group + negative control group.

The proliferative performance of P0-6 BMSCs in the experimental group, negative control group, and blank control group was discovered at 4, 8, 12, 16, 20, and 24 hours, by testing the living cells for their OD value. As shown in Figure 3A–3C, the results showed that the experimental group's OD value peaked at eight hours after E16 transplantation on E19, then progressively declined, and was substantially differing from the blank control and negative control groups (*P<*0.05). The OD peak timing and value among the negative control and blank control, however, were not significantly different.

### pSTAT3 and STAT3 expression analysis in each group following BMSCs transplantation

**Figure 4:**
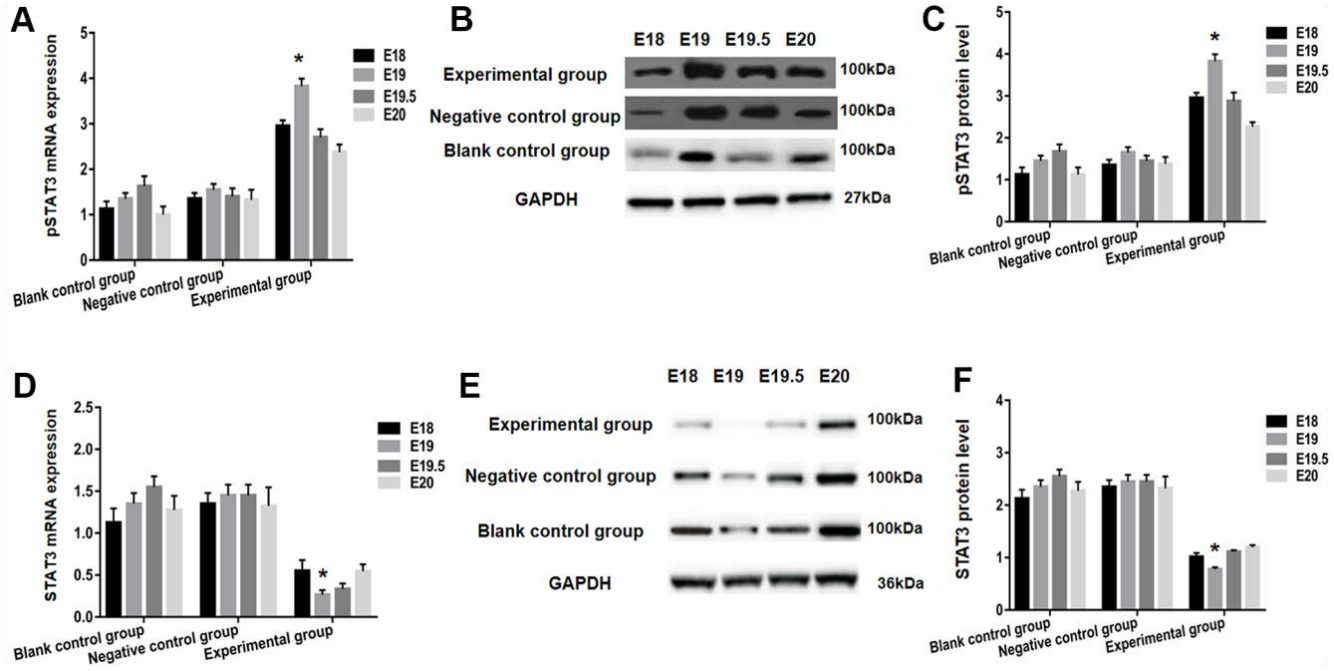
**STAT3 and pSTAT3 expression in the spinal cord of each group after BMSCs transplantation using RT-PCR and western blot analysis.** The pSTAT3 mRNA expression was detected utilizing RT-qPCR (**A**), and protein expression using western blot analysis (**B**, **C**). Both pSTAT3 mRNA and protein levels were significantly increased within the experimental group from E18 to E20, with the peak being at E19. In addition, expression of STAT3 mRNA was analyzed by RT-qPCR (**D**). Protein levels were assessed using western blot (**E**, **F**). The expression of STAT3 mRNA and protein levels in the experimental group gradually decreased with embryonic development between E18 and E19; E19 dropped down to bottom. * *P* <0.05 versus blank control group + negative control group.

On E18, E19, E19.5, and E20, the experimental group's developmental changes in the expression of pSTAT3 following the transplantation of P1-3 BMSCs were substantially differing from the negative control and blank control groups, and were particularly notable at embryonic day 19 (E19). From E18 to E20, the experimental group's pSTAT3 mRNA and protein levels rose substantially, with E19 being the highest (Figure 4A–4C). In the experimental group, between E18 and E19, the expressions of STAT3 mRNA and the levels of the protein steadily declined with embryo development, with the lowest being on E19. The levels of STAT3 and pSTAT3 mRNA and protein in the groups of negative control and blank control remained stable from E18 to E20, which is statistically lower in comparison to the experimental group (*P<* 0.05) as illustrated in Figure 4D–4F.

### Nestin, NF, NSE, GFAP expression analysis in each group following BMSCs transplantation

**Figure 5:**
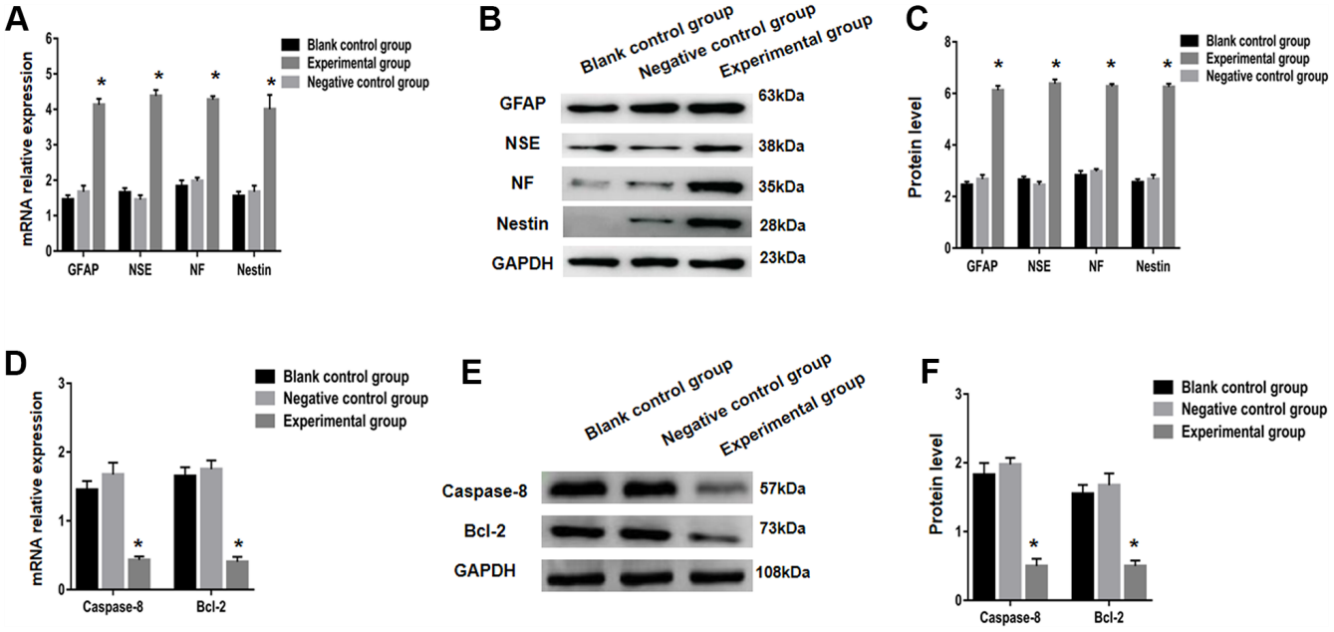
**Nerve markers (GFAP, NSE, NF and nestin) and apoptosis related factors (caspase-8 and bcl-2) expressions were tested using real-time PCR and Western blot.** (**A**) Expression of GFAP, NSE, NF and nestin in the blank control group, negative control group and the experimental group were examined using RT-qPCR. GFAP, NSE, NF and nestin protein expression was analyzed using western blot analysis (**B**, **C**). Relative mRNA expression of apoptosis related factors (caspase-8 and bcl-2) was determined using RT-qPCR (**D**) and protein expression using western blot analysis (**E**, **F**). * *P <* 0.05 versus blank control group + negative control group.

When P1-3 BMSCs were transplanted on E16, the relative expression of GFAP mRNA was considerably different in all three studied groups, namely the experimental, negative control, and blank control. On E19 transplanted fetuses, the levels of GFAP mRNA in the experimental group were substantially greater in comparison to negative control and blank control group (*P<*0.05). Parallelly, the comparative expressions of NF, NSE, and nestin mRNA in individual groups were essentially the same as GFAP (Figure 5A). Furthermore, Western Blot protein expression of NF, NSE, GFAP, and nestin was similar to real-time PCR (Figure 5B, 5C).

### Bcl-2 and caspase-8 expression analysis in each group following BMSCs transplantation

Apoptosis genes like bcl-2 and caspase-8 play a key role in spina bifida aperta development. Then, using STAT3 overexpression plasmid transfection, bcl-2 and caspase-8 expressions, we looked at apoptosis in sacral spinal cord tissue following BMSC transplantation. The levels of both protein and mRNA of bcl-2 and caspase-8 were considerably lower in the experimental group compared to the other two groups at P1-3 BMSCs on E16 transplantation, with a statistical significance on E19. (Figure 5D–5F).

## DISCUSSION

It is a highly dynamic process in mammalian embryonic development that is tightly linked to embryonic cells division, growth, differentiation, and death, and may be disturbed by a variety of signaling pathways. The STAT3 pathway controls a number of embryonic development pathways and is involved in aberrant embryonic development, which can be affected by valproic acid [16]. STAT3 also facilitates gene transcription in response to intermediary criteria including IL-6, caspase-8, EGF, and bcl-2. These factors, on the other hand, control cell proliferation, survival, and death throughout embryogenesis. STAT3 is a non-phosphorylated monomer that is inactive *in vivo*. STAT3 will undergo phosphorylation followed by homologous or heterodimerization after being activated by these cytokines. STAT3 then reaches the nucleus and binds to particular nucleotide sequences, triggering the production of a variety of downstream target genes [17]*.* According to our findings, the levels of pSTAT3 in mRNA and protein within the experimental group grew considerably from E18 to E20, with E19 being the highest. Between E18 and E19, the levels of STAT3 protein and mRNA expression in the experimental group steadily declined with embryonic development, with E19 being the lowest. This research shows that STAT3 undergoes phosphorylation throughout the transplantation process and is then transformed to pSTAT3 on E19, thereby potentially activating downstream target genes as well as enhancing BMSC trans-differentiation.

Our findings revealed that BMSCs coupled with STAT3 overexpression plasmid transfection are able to significantly increase cell survival and purity. In the experimental group, cell transplantation from P1 to P3 on E16 with medium injection had a greater therapeutic effect than the other two groups. The STAT3 pathway is extremely important in the differentiation and migration of BMSCs [18]. Because BMSCs have a high capacity for self-renewal and pluripotency, they can be readily converted into fat cells, cartilage cells, bone cells, endothelium cells, myocytes, nerve cells, and other types of cells given the right conditions [19]. SBA transplantation from BMSCs can help with a number of bone and neuronal problems. BMSCs are also simple to collect, operate, grow *in vitro*, and purify and may be passed through several times to preserve differentiation potential. In addition, the use of BMSCs does not result in immunological rejection. Recent research has found that BMSCs may survive spinal cord injury and develop into astrocytes and neurons, reducing the harm resulting from nerve function loss [20]. Consequently, BMSCs may be employed as cells for tissue engineering, and BMSC transplantation for SBA therapy is predicted to be an effective and innovative approach for treating nervous system abnormalities. The goal of surgical spinal cord repair for early in utero therapy of rat embryos with SBA was to prevent future deformity from worsening. STAT3 can interact with various pathways of signaling that are expressed and active during neural circuit formation, such as MAPK and PI32K, and is involved in the stem cells' differentiation in the neural tube epithelium [21]. Peripherin is a neuron-specific intermediate filament protein that is expressed in all motor, sensory, and peripheral neurons and possesses a STAT3 site of binding in its promoter [22]. Under the influence of LIF and IL-6, STAT3 exhibits transcriptional activity, indicating that it also has a regulatory role in peripheral neurons [23]. Activation of STAT3 is able to particularly mediate the directional differentiation of type II astrocytes as reported by Aberg M et al. [24]. As a result, our findings show that the JAK/STAT signaling pathway, which may be triggered by cytokines, is a key factor in regulating cell differentiation direction throughout mammalian development. Despite the fact that STAT3 is known as the most essential regulatory genes in the nervous system's advancement, the rate of survival for embryos following gene deletion is extremely low *in vivo*, limiting STAT3 study into the regulatory procedure and molecular strategy [25].

Our findings show that the experimental group's OD value peaked at eight hours following the transplantation. Next, progressively declined, which was substantially diverse from the negative control and blank control groups. Transfection of STAT3 overexpression plasmid into neural cells and BMSCs, on the one hand, accelerates transformation eight hours after transplantation. However, it has the potential to boost cell survival and proliferation. The success of BMSCs *in vitro* culture has led to the development of a useful experimental model for the investigation of essential biological features like proliferation, differentiation, and migration in recent years [26]. As a result, no studies have been done on the impact of the partial activation of STAT3 on BMSC differentiation and proliferation *in vitro*. The preservation of pluripotency and the undifferentiated condition of embryonic stem cells is linked to the JAK/STAT3 signaling pathway [27]. Upon treatment with LIF, stem cells that overexpress negative STAT3 lose their capacity to self-renew and differentiate, indicating that STAT3 is linked to the proliferation of neural stem cells [28]. Furthermore, some investigations have illustrated that STAT3 is vital for the neural precursor cells differentiation *in vivo*, interacting with other pathways of signaling, for instance, MAPK, and caspase-8, and regulating differentiation alongside neuromodulatory cytokine (NF), CT1 (cardiotrophin), leukemia inhibitory factor (LIF), ciliary neurotrophic factor (CNTF), and oncostatin M [29]. As STAT3 inhibitors, the SOCS family behave as a negative feedback protein in this process, inhibiting the activity of JAK. Furthermore, STAT3 possesses a site of binding that responds quickly to the promoter of glial fibrillary acidic protein (GFAP) and stimulates GFAP transcription in the CNTF-induced differentiation of cortical precursor cells into glial cells. As a result, it's thought that STAT3 is strongly linked to astrocyte differentiation [30]. Further research shows that STAT3 forms a complex with Smad1 and p300 called STAT3/Smad1/p300 via the synergistic processing of bone morphogenetic protein 2 (BMP-2) and LIF that mediates NF, NSE, and nestin promoter activation, and causes the differentiation of primary embryonic neural precursor cells into astrocytes [31]. STAT3 primarily causes neural stem cells to develop into astrocytes, according to the data shown above.

Our findings also demonstrate that the experimental group's protein and mRNA expression of GFAP, NF, NSE, and nestin are considerably greater than the other two groups. STAT3 appears to be involved in BMSCs differentiation into osteocytes, muscle cells, and neuroglia cells, according to these findings. In PC12 cells, neurite development is mediated through the STAT3 signaling pathway and MAP kinase. *In vitro*, STAT3 activation can inhibit neurite elongation in PC12 cells that use the Ras-MAPK pathway and increase sympathetic neuron dendrite contraction [32]. The ability of STAT3 to control neurite development has been established, which corroborates quite well with our findings.

Meanwhile, on E19, the levels of both protein and mRNA of bcl-2 and caspase-8 were remarkably lower in the experimental group in comparison to the negative control and blank control groups in our study. The findings suggest that BMSC transplantation following STAT3 overexpression plasmid transfection may aid in reducing apoptosis in the spinal column. Caspase-8 is a fundamental modulator of death receptor-induced apoptotic pathways and is an essential starter in the caspase cascade. Evidence shows that caspase-8 has become an indispensable part of Fas-mediated apoptotic pathways [33]. Activated caspase-8 is firstly to cleave, and then activates downstream signal of caspases, thereby eventually resulting in apoptosis. Bcl-2 and caspase-8 also activate other related apoptotic pathways through the mediated regulation of Bid, facilitates cytochrome C released from mitochondria, and the downstream caspase-9 activation. Caspases-8 participates in apoptotic signaling through a variety of death receptors, comprising TNF-related apoptosis-inducing ligand (TRAIL) receptors, death receptor 3 (DR3), and tumor necrosis factor receptor type 1 (TNFR1), besides Fas. Bcl-2 and Caspase-8 are clearly key mediators for activating different apoptotic pathways [34]. The bcl-2 and caspase-8-mediated apoptotic pathway is activated within BMSC transplantation following STAT3 overexpression plasmid transfection and hence needs to be further investigated. Briefly, the obtained findings provide the notion that transfection of BMSCs with a recombinant lentivirus-mediated STAT3 overexpression plasmid is able to increase the efficacy of neural cell transformation and supply fresh seed cells for treating congenital spina bifida aperta.

## MATERIALS AND METHODS

### Ethics approval

The animal experiment protocols were carried out in accordance with laboratory norms and were authorized by Harbin Medical University's ethical committee.

### Animal models

For the study, 10-12 week old Wistar adult rats weighing 250-300 g were chosen. The rats were obtained from Harbin Medical University's Animal Research Center (Harbin, China). Male rats from the same strain were mated with virgin female rats. E0 was the date when the vaginal plug was discovered. On E10, a single intragastric injection of retinoic acid (140 mg/kg body weight; Sigma) was used to produce spina bifida aperta, as previously described [35].

### Isolation and transfection of BMSCs

The femurs of four-week-old Wistar rats were used to obtain the cells. 20 mL DMEM/F12 medium and 10% fetal bovine serum were used to flush the rat bone marrow (FBS). For a primary generation, the suspension of cells was separated as BMSCs using the bone marrow adherence technique (P0). The cells were then planted and allowed to develop within an incubator of tissue cultivation at 37° C and 5% CO_2_. To get the appropriate dosage, cells were passaged for five to seven days till passage 6 (P6). Prior to transfection, P6 BMSCs were plated in a six-well plate and allowed to develop to 90% confluence (approximately 1×10^5^ cells/well). Transfection of the BMSCs was carried out with a recombinant lentivirus and STAT3 overexpression plasmid (SinoGenoMax.Co., Ltd, Beijing, China) (100 pfu/1cell) 24 hours before transplantation. pHBLV-U6-ZsGreen-PGK-Puro fragment was selected as the cloning vector. The clone entry point is xhoi/BamHI. After the STAT3 plasmid synthesis, the sequence accuracy was further confirmed by verification of the plasmid supplier. The titer of lentivirus was maintained at 3×10^7^, which was used as a vector to transfect STAT3 overexpression plasmid into BMSCs (MOI = 300-500), a suspension of approximately 20000 BMSCs cells /μL was employed for transplantation.

### BMSCs identification via flow cytometry

Flow cytometry was used to examine the surface proteins CD45, CD34, CD90, and CD73. The subsequent experiments are scientifically supported by the extraction, identification as well as purity characterizations for BMSCs.

### Viability of BMSCs was ascertained through CCK-8

Firstly, we carried out the cell count. Cells were seeded at a density of at least 5× 10^4^ cells per well onto four 96-well plates. Two duplicate wells were established and cultured in the cell incubator at 37° C in parallel. Then, at 4, 8, 12, 16, 20, 24, 48, and 72 hours, 10 μl of the solution of CCK-8 (TaKaRa Bio Inc, Japan) was put in individual wells. At 450 nm, the OD magnitude was ascertained.

### Transplantation procedure and animal group

Overall, 72 female Wistar rats were analyzed in the current work. Animals had uninterrupted accessibility to water and food during the experiments. All operations were performed under anesthesia using 40 mg/kg pentobarbital sodium (Sigma) and 0.25 mg/mL atropine (Sigma) intraperitoneally on three separate embryonic days (E15, E16, and E17), as previously described [35]. To reveal the uterine horns, the pregnant rats were then subjected to a midline laparotomy. The uterus was also wrapped with drippy gauze saturated with atropine and warm physiologic saline (0.1 mg/kg) to alleviate hysterospasm before the intraperitoneal injection was finished. Under the assistance of a microscope, we determined the precise location of the lumbosacral spine in fetal rats via the wall of the uterus. A tiny incision and a 7-0 nylon purse-string suture were then used to construct the uterine wall. The amniotic sac was discovered to open, exposing the abnormality in the spinal cord tissue. A micropipette having an internal tip diameter equivalent to 100 μm attached to a Hamilton syringe (experimental group) was used to inject 0.2 μL BMSCs with STAT3 overexpression plasmid transfection in each of the defected regions. Micropipettes were injected into borosilicate glass capillaries (model GD-1; Narishige Scientific Instruments, Tokyo, Japan) and a micropipette puller was used (model PB-7; Narishige Scientific Instruments, Tokyo, Japan). The unborn rats were placed back into the womb once the BMSCs injection was completed, and the uterine incision was closed. The fetal rats were split into three groups based on the sorts of BMSC injections they received. These groups contained the identical number of BMSCs without recombinant lentivirus transfection (blank control group) and just recombinant lentivirus transfection without STAT3 overexpression plasmid (negative control group). To keep the unborn rats alive, we injected three to four fetuses into each dam on average. The pregnant rats were sent back to their home cage after recovering from the anesthetic in approximately an hour. The pregnant rats were re-anesthetized as needed on E18, E19, E19.5, and E20, and the transplanted fetal rats were removed through cesarean section. External abnormalities in each fetus were checked.

### Real-time polymerase chain reaction assessment

Isolation of Total RNA and cDNA was executed conforming to the instructions of the manufacturer. 20 μL reaction volume of the All-in-one qPCR Mix (TaKaRa Bio Inc) includes 1 μL of cDNA, 10 μL of 2×All-in-one qPCR Mix (TaKaRa Bio Inc), 1 μL of 2 mmol/L reverse primers, 1 μL of 2 mmol/L forward primers, and 6 μL of nuclease-free water. Denaturation at 95° C for 10 minutes, succeeded by 40 cycles of 10 seconds at 95° C, 20 seconds at 60° C, and 15 seconds at 72° C. The primers of Glyceraldehyde 3-phosphate dehydrogenase (GAPDH, TaKaRa Bio Inc) were utilized for the estimation of the amount of GAPDH cDNA in each sample for normalization. Table 1 lists the primers employed in this research. Determination of the comparative fold-change in the target gene cDNA was carried out via the use of the 2^−ΔΔCt^ approach.

**Table 1:** Primers used in the study.

**Genes**	**Primer sequences**	**Annealing** **temperature (° C)**	**PCR product (bp)**
GAPDH	sense:5′-TCGCCAGCCGAGCCACAT-3′	60	149
	anti-sense: 5′-GGAACATGTAAACCATGTAGTTG-3′		
STAT3	sense:5′- GCCAGAGAGCCAGGAGCA -3′	60	135
	anti-sense:5′-ACACAGATAAACTTGGTCTTCAGGTATG -3′		
pSTAT3	sense:5′- CGAACTTCATTCAACAATGA -3′	60	109
	anti-sense: 5′- GTTTAGCCTAAGAGGTATAG -3′		
GFAP	sense:5′- ATACAGATATAGGGATTACA-3′	60	98
	anti-sense: 5′- CTTGGATTCGGACCAGTTAG -3′		
NSE	sense:5′- AGATATAGGGATTACAGGATT-3′	60	91
	anti-sense: 5′- ACCAGTTAATTCGGATCTGAC -3′		
NF	sense:5′- CCGCAGATTACTGATTCAGG-3′	60	101
	anti-sense: 5′- CTTGGCCAGTTCTAAACGGA -3′		
nestin	sense:5′- TTCAGGCAGAGAGCCTTACT-3′	60	112
	anti-sense: 5′- AGTTCTTGGAACTACGGACC -3′		
caspase-8	sense:5′- AAATCTGCAAACGAGAATGC -3′	60	118
	anti-sense: 5′- CACAGGATGTTCCCCAGATT -3′		
bcl-2	sense:5′- AGAATTCCTTAGAGATTCTT -3′	60	127
	anti-sense: 5′- CAACGATACTATTACTATACGA-3′		

### Western blotting assessment

Western blotting was used for determining the protein concentration in the tissues of the spinal cord. In brief, we lyse tissues from various groups with Radio-Immunoprecipitation assay lysis buffer (Beyotime Bio Inc, Shanghai, China) and measure protein quantities with the BCA protein evaluation kit (Beyotime Bio Inc). Later, using a sodium dodecyl sulfate-polyacrylamide gel electrophoresis (Beyotime Bio Inc), protein specimens of identical amounts were separated and moved to membranes fabricated from polyvinylidene difluoride (Beyotime Bio Inc). Following blocking with 5% non-fat dry milk, the membranes were treated at 4° C during the night hours with primary antibodies. GAPDH (Santa Cruz Bio Inc, USA), caspase-8 (Santa Cruz Bio Inc, USA) and STAT3 (Santa Cruz Bio Inc, USA) were the set of primary antibodies employed in the work. Then, to bind with the horseradish peroxidase, the appropriate secondary antibodies were added. The blotting findings were seen using an ECL detection reagent provided by 7 Sea Biotech, Shanghai, China. A Gel-Pro-Analyzer (Media Cybernetics, USA) was implemented to quantify the data.

### Statistical assessment

The SPSS version 20.0 computer program (SPSS, Inc, USA) was implemented to conduct all statistical analyses. The unpaired Student t-test was employed for determining the differences among just two sets of values. The differences between three or more groups were then scrutinized by employing a one-way assessment of variance. The data comes from at least three separate experiments and is provided as a mean and standard deviation. Statistical significance was described as a *P*-value *<* 0.05.

### Ethics approval

The study was granted approval by the ethics committee at Harbin medical University of technology committee.

### Availability of data and materials

The datasets used and/or analyzed during this study are available from the corresponding author upon reasonable request.
